# Upcycled Orange Peel Ingredients for Gastrointestinal and Cardiometabolic Health: A Scoping Review and Market Perspectives

**DOI:** 10.3390/nu18071126

**Published:** 2026-03-31

**Authors:** Ana A. Vilas-Boas, Marta Correia, Débora A. Campos, Manuela Pintado

**Affiliations:** CBQF—Centro de Biotecnologia e Química Fina—Laboratório Associado, Escola Superior de Biotecnologia, Universidade Católica Portuguesa, Rua Arquiteto Lobão Vital 172, 4200-374 Porto, Portugal; avboas@ucp.pt (A.A.V.-B.); mmcorreia@ucp.pt (M.C.); dcampos@ucp.pt (D.A.C.)

**Keywords:** hesperidin, pectin, gut microbiota, metabolic disorders, nutraceutical

## Abstract

**Background:** Orange peels (OP) are rich in flavonoids, pectin, essential oils, and carotenoids that can be upcycled into functional ingredients. These bioactive compounds (BCs) have been increasingly associated with beneficial effects on gastrointestinal (GI) and cardiometabolic health. This scoping review aimed to systematically map available evidence and synthesize reported GI and cardiometabolic health outcomes of upcycled OP ingredients. **Methods:** Conducted in accordance with PRISMA-ScR guidelines, the literature search was performed in the Scopus database and ClinicalTrials.gov for studies published between 2014–2025 using a predefined Boolean search query. After screening, 84 studies and 14 completed clinical trials met the inclusion criteria. **Results:** The mapped evidence spans mechanistic (in vitro), preclinical, and clinical studies. Preclinical studies report that flavonoids, pectin, and essential oils modulate gut microbiota composition, enhance intestinal barrier integrity, and improve glycemic, lipid, and inflammatory parameters through mechanisms involving short-chain fatty acid production, antioxidant activity, and modulation of key inflammatory pathways. Clinical studies, predominantly investigating hesperidin-rich and fiber-rich extracts, report improvements in postprandial glycemic response, lipid profiles, blood pressure, and selected microbiota-related markers. **Conclusions:** Upcycled OP ingredients show promising biological activities relevant to cardiometabolic health and gut modulation, particularly in mechanistic and preclinical models. However, the evidence base remains largely dominated by in vitro and animal studies, with limited and heterogeneous clinical data. Key gaps include the underrepresentation of pectin and carotenoids in human trials and the absence of standardized, long-term intervention studies. Future research should prioritize well-designed clinical trials and investigate potential synergistic interactions among OP-derived bioactive fractions to support their translational application.

## 1. Introduction

The global nutraceutical products market was valued at USD 267.4 billion in 2019 and is projected to reach USD 404.8 billion by 2025, growing at a Compound Annual Growth Rate (CAGR) of 7.2%, driven by the rising prevalence of chronic diseases such as obesity, type 2 diabetes, cardiovascular diseases, and cancer, the increasing geriatric population, and growing consumer awareness of the role of diet in health promotion [1]. In response, the nutraceutical and functional food sectors have rapidly expanded, with consumers increasingly seeking natural, science-based products that support disease prevention and promote overall vitality [2].

*Citrus sinensis* (L.) Osbeck, commonly known as sweet orange, is one of the most widely consumed citrus fruits worldwide and represents a major raw material for citrus juice production [3]. The industrial processing of sweet oranges generates substantial quantities of by-products, particularly orange peels (OPs), which represent both an environmental challenge and an opportunity for developing value-added ingredients within circular bioeconomy strategies [4]. OPs are a rich source of bioactive compounds (BCs) such as phenolic compounds, essential oils, carotenoids and pectin. These BCs have been associated with multiple health-promoting properties, such as antioxidant, anti-inflammatory, antimicrobial, and prebiotic effects, highlighting the potential of upcycled OP ingredients for the development of functional foods, supplements and nutraceuticals. A recent bibliometric analysis [5] from our group revealed an exponential increase in scientific publications on the valorization of OP to develop functional ingredients over the last decade, with a clear shift from environmental applications to health-related uses. Phenolic compounds and pectin emerged as the most studied BC targets, reflecting their antioxidant, anti-inflammatory, and prebiotic properties [6]. Recent trends highlight growing interest in biorefinery, green extractions, polyphenols, fibers and nutraceutical applications, indicating a transition toward health-related applications and nutraceutical potential. Despite the rise in publications, there is a marked absence of research on gut modulation, cardiometabolic effects, and human clinical trials. These findings underscore the need for a comprehensive synthesis of mechanistic evidence and market applications of upcycled OP ingredients.

The bioactivities reported for OP are not independent but converge within the gut–cardio–metabolic axis, a key mechanism through which BCs exert systemic benefits. GI and cardiometabolic health are closely interconnected via shared pathophysiological pathways, including chronic inflammation, oxidative stress, and gut microbiota regulation [6,7]. The regulation of cardiometabolic homeostasis involves intricate crosstalk between the GI tract and peripheral organs, including the brain, liver, pancreas, adipose tissue, and skeletal muscle, all of which communicate via nutrient-derived signals, gut hormones, and microbial metabolites [8]. This interorgan axis plays an important role in the pathogenesis and prevention of metabolic disorders, supporting the relevance of BCs that act on both gut and systemic targets.

Flavonoids from OP, particularly flavanones and polymethoxyflavones (PMFs), have demonstrated a broad spectrum of bioactivities, including anti-inflammatory, antioxidant, anti-cancer, anti-obesity, anti-atherosclerosis, and anti-diabetic [8,9]. Due to their good antioxidant and anti-inflammatory properties, these flavonoids have been investigated for their protective effect against GI and cardiometabolic diseases for years. However, recent studies highlighted that OP flavonoids can modulate gut microbiota composition by selectively enhancing the beneficial taxa such as *Akkermansia muciniphila* and *Bacteroides ovatus*, while reducing the Firmicutes-to-Bacteroidetes ratio, a known dysbiosis marker in metabolic disorders. This positive modulation of the microbiota, along with microbial metabolism of flavonoids into phenolic acids and other bioactive metabolites, such as short-chain fatty acids (SCFAs), contributes to improved gut barrier function and systemic benefits, particularly in glucose and lipid homeostasis [10]. Due to these properties, OP flavonoids, particularly for their ability to selectively modulate gut microbiota composition, enhance the production of SCFAs, and reduce intestinal inflammation, have been proposed to function as a distinct class of prebiotic-like compounds. This concept has been commercially trademarked under the name Flavobiotics^TM^ by a nutraceutical brand to describe citrus-derived flavonoids with prebiotic activity.

On the other hand, pectin is well recognized for multiple health benefits, mainly through its prebiotic effects [11]. Once fermented by gut microbiota, pectin promotes the production of SCFAs, which modulate glucose metabolism, improve intestinal barrier integrity, and reduce inflammation by interacting with receptors such as GPR41, GPR 43, and TLRs [12,13]. Additionally, pectin enhances mucosal immunity and gut epithelial repair. These effects have been linked to the modulation of bile acids and inflammatory cytokines, contributing to improved cardiometabolic outcomes. However, most studies use citrus pectin instead of pectin derived from OP, and the clinical data validating these benefits remain lacking. Beyond pectin and flavonoids, other bioactive fractions obtained through OP valorization, such as essential oils (EO) and carotenoids, have demonstrated antioxidant, antimicrobial, and potential anti-inflammatory properties in preliminary in vitro studies.

Nevertheless, evidence in these areas remains scarce and highly fragmented, with most reports limited to in vitro and animal-studies assays and lacking systematic evaluation of their mechanisms or health outcomes. The absence of robust clinical trials and comprehensive reviews of upcycled OP ingredients underscores the need for a structured synthesis to clarify their roles in GI and cardiometabolic health. Notably, no comprehensive scoping review has yet integrated mechanistic, preclinical, and clinical evidence on upcycled OP ingredients specifically within the context of GI and cardiometabolic health.

This scoping review aims to systematically map and critically synthesize the current evidence on the health-promoting effects of upcycled OP ingredients on GI and cardiometabolic health. Given the heterogeneity of available studies and the emerging nature of this topic, a scoping review approach was selected to provide a comprehensive overview. Evidence from in vitro, in vivo, and human clinical studies was examined to explore the impact of key BCs, including flavonoids, EO, and pectin, on gut microbial composition, intestinal barrier integrity, metabolic markers, and pathways related to inflammation and oxidative stress. In addition, this review uniquely assesses the commercial landscape of OP-based nutraceuticals, summarizing ingredients currently marketed with clinically validated benefits. By identifying research gaps and outlining future directions, this work highlights the potential of OP upcycling within circular bioeconomy strategies and sustainable nutrition frameworks to improve population health.

## 2. Research Methodology

This scoping review was conducted and reported in accordance with the PRISMA Extension for Scoping Reviews guidelines to ensure methodological transparency and reproducibility [14]. No protocol was registered for this review. In January 2024, we conducted the literature search across three databases, Scopus, Web of Science (WoS) and PubMed, covering publications from 2014 to 2023. The search strategy followed a two-step Boolean approach. First, a core query was applied to identify studies specifically referring to OP, targeting article title containing the keywords orange peel, orange pomace, orange by-product, orange byproduct or orange waste (query: TITLE (“orange peel*” OR “orange pomace” OR “orange by-product*” OR “orange byproduct*” OR “orange waste*”) AND PUBYEAR > 2013 AND PUBYEAR < 2024). A cross-check of the results revealed that PubMed returned approximately 20% fewer results than the other two databases, and all records retrieved from WoS were also indexed in Scopus. Given this overlap and Scopus’s broader coverage, the final analysis was based exclusively on Scopus output to avoid duplication and ensure consistency. Filters were applied to include only peer-reviewed articles published in English. Subsequently, this core query was combined using the Boolean operator AND with thematic keyword groups related to health outcomes, as detailed in Appendix A (Table A1). This approach ensured that all retrieved records explicitly referred to upcycled OP ingredients while capturing a broad range of potential biological activities.

**Figure 1:**
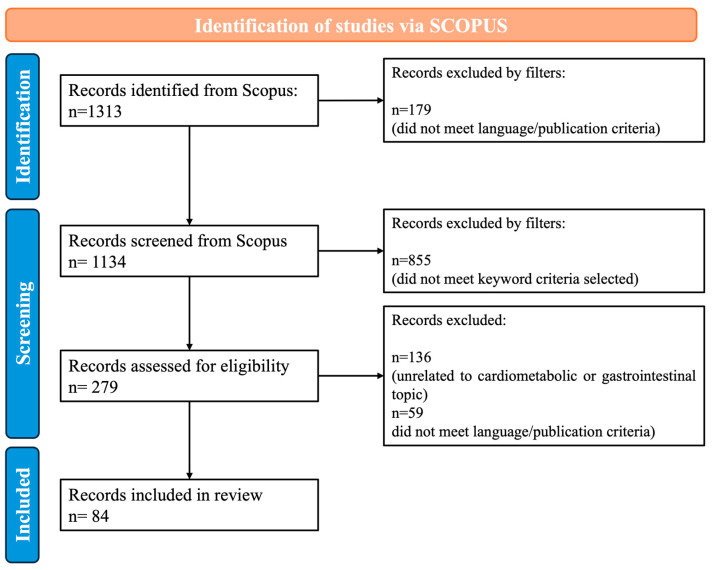
PRISMA-ScR flow diagram of the study selection process.

Two reviewers independently screened titles and abstracts, removed duplicates, and assessed full texts for eligibility (studies investigating ingredients obtained from synthetic sources or other citrus materials not specifically derived from OP were excluded). Discrepancies were resolved by consensus. After full-text assessment, studies focusing on cardiometabolic and GI health were included in the review. To ensure currency of the evidence base, the search was subsequently updated in March 2026 to include publications from 2024 and 2025, using the same query, database, and eligibility criteria as the original search. The study selection process is summarized in Figure 1.

For human clinical trials, we searched both ClinicalTrials.gov and Scopus in January 2026. Clinical studies were eligible for inclusion if they: (i) were conducted in human participants; (ii) investigated an ingredient derived exclusively from OP (e.g., extracts, isolated flavonoids, pectin, essential oils, or carotenoids); (iii) reported outcomes related to GI or cardiometabolic health, and (iv) results were published in peer-reviewed journals. Data extraction was performed independently by two reviewers. For each included study, the following information was systematically collected: study design, population characteristics and number of participants, ingredient used, dose and intervention duration, and outcomes assessed. Discrepancies between reviewers were resolved by consensus. As this is a scoping review, no formal risk-of-bias assessment of the included studies was conducted, as methodological appraisal is generally not applicable to this review typology [15,16].

## 3. Results and Discussion

### 3.1. Publication Trends on Health Effects of Upcycled Orange Peel Ingredients

As scoping reviews aim to map the extent, characteristics, and evolution of the available literature on a given topic, an analysis of publication trends was conducted to examine how research on upcycled OP ingredients and their potential health applications have evolved over time. This overview helps contextualize the current evidence base, identify emerging research directions, and highlight existing knowledge gaps in this field.

**Figure 2:**
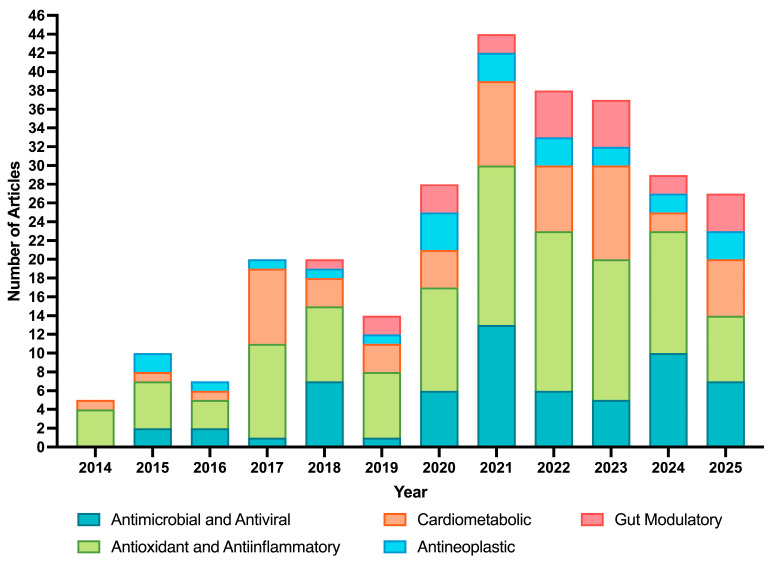
Annual number of publications (2014–2025) on upcycled OP ingredients categorized by health-related bioactivities.

Figure 2 illustrates the distribution of publications related to the health effects of upcycled OP ingredients (*n* = 284) across major bioactivity categories from 2014 to 2026. A pronounced increase in publications after 2020 is evident across all categories, reflecting growing scientific interest in the valorization of OP and the expanding nutraceutical market.

Research has predominantly focused on antioxidant, anti-inflammatory, antimicrobial, and antiviral properties, which are relevant for addressing oxidative stress and inflammation-related disorders. The high volume of publications in these categories partly reflects the widespread use of standardized, high-throughput in vitro assays such as, DPPH, ABTS, and ORAC, which are technically accessible and widely adopted for initial bioactivity screening. However, it is well recognized that these chemical assays have limited physiological relevance, as they do not account for bioavailability, bioaccessibility, or the complexity of human metabolism, and may therefore not reliably predict antioxidant efficacy in vivo. Notably, the antioxidant and anti-inflammatory category consistently represented the largest proportion of publications across all years, suggesting that despite diversification into cardiometabolic and gut-related research, the field has yet to fully transition toward more complex and physiologically relevant experimental models.

Several studies have investigated hesperidin, the major flavonoid in OP, for its potential as an inhibitor of SARS-CoV-2, particularly during the COVID-19 pandemic [17]. Importantly, 2021 represented the peak year of publication output (*n* ≈ 44), followed by a gradual decline through 2024–2025 (*n* ≈ 27–29). Interest in the antineoplastic (anticancer) potential of upcycled OP ingredients has also increased since 2020. Although this category remains smaller than antioxidant or antimicrobial research, its growth highlights the increasing exploration of polyphenol-rich extracts as complementary strategies for disease prevention.

Cardiometabolic and gut-modulatory research categories have increased over time, particularly since 2021, encompassing studies on cardiovascular health, glycemic control, obesity, and gut microbiota modulation. Nevertheless, despite growing recognition of the gut–cardio–metabolic axis as a central mechanism through which OP bioactives exert systemic effects, gut-modulatory research consistently represented one of the smallest publication categories throughout the entire period analyzed. This bibliometric observation directly corroborates the clinical gaps identified in this review, particularly the underrepresentation of human studies evaluating pectin and essential oil-derived ingredients in the context of microbiota modulation and intestinal health. Within this context, the role of phenolic compounds as gut microbiota modulators has gained increasing attention, with growing evidence suggesting that polyphenols reaching the colon intact can selectively promote beneficial bacteria, suppress pathogens, and stimulate the production of health-promoting metabolites, with direct implications for conditions such as obesity and type 2 diabetes.

Overall, this research trajectory aligns with the United Nations Sustainable Development Goal 3 (Good Health and Well-being), which seeks to reduce non-communicable diseases and promote global health through innovative, sustainable strategies.

### 3.2. Impact of Upcycled OP Ingredients on Cardiometabolic and Gastrointestinal Health: Evidence from In Vitro, In Vivo, and Clinical Studies

Cardiometabolic health encompasses interconnected processes that impact cardiovascular and metabolic functions, including blood pressure regulation, glucose and lipid metabolism, and inflammation management [17]. These alterations contribute to the development of major chronic diseases such as hypertension, atherosclerosis, type 2 diabetes, obesity, and non-alcoholic fatty liver disease (NAFLD). A clinical condition that exemplifies this overlap is metabolic syndrome (MetS), defined by the co-occurrence of at least three metabolic risk factors including abdominal obesity, insulin resistance, dysglycemia, atherogenic dyslipidemia (elevated triglycerides and/or low HDL-cholesterol), and hypertension [18]. MetS presents a significant public health concern, substantially raising the risk of developing cardiovascular disease and type 2 diabetes, by up to five times. With its global prevalence steadily increasing, largely due to sedentary lifestyles and poor dietary habits, there is a growing scientific interest in natural bioactive ingredients that can modulate these metabolic disturbances and provide preventive health benefits [19].

Cardiovascular diseases are the primary cause of death globally [20], reflecting the impact of metabolic risk factors, including high blood pressure, high blood sugar, high body mass index, and elevated low-density lipoprotein (LDL) cholesterol. According to the World Health Organization, these risk factors, closely interlinked with modern lifestyle changes, have driven a sharp rise in metabolic conditions like type 2 diabetes and obesity across all income levels over the past 30 years [21]. In 2021, 537 million people—about 10.5% of the global population—were affected by diabetes, with related healthcare costs reaching $966 billion [22]. Recent statistics reveal that 2.5 billion adults and 37 million children under five are overweight, including 1.05 billion adults living with obesity. Together, cardiometabolic disorders create a complex health burden, underscoring the need for preventive strategies to address interconnected health risks on a global scale. These overlapping risks also perpetuate a cycle of dysfunction that adversely affects GI health, highlighting its close relationship.

GI health encompasses the proper functioning of the digestive system, including the digestion and absorption of nutrients, maintaining a balanced gut microbiota, and preventing disorders such as inflammatory bowel disease (IBD) and cancers [23,24]. The gut microbiota is highlighted as an important component of GI health. Structural GI conditions, such as colorectal cancer and IBD, involve abnormal bowel changes that may require surgical intervention, with IBD affecting 0.2% of Europeans, which includes Crohn’s disease and ulcerative colitis [25]. IBD is positively correlated with intestinal cancer development [26]. Colorectal cancer is the third most common cancer globally and the second leading cause of cancer-related deaths. In 2022, the European Cancer Information System reported 362 million new colorectal cancer cases and 161 million deaths in Europe, with a total economic burden of €19 billion, projected to rise further.

**Figure 3:**
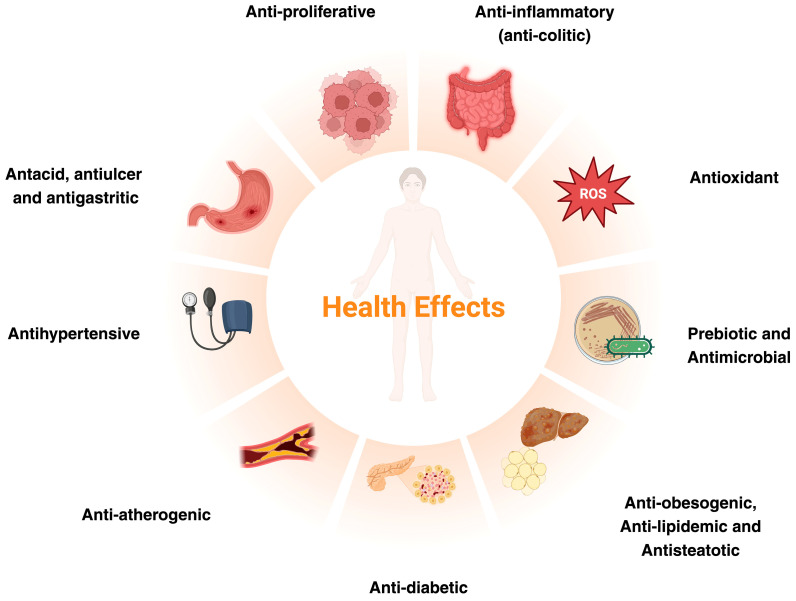
The main health benefits of upcycled OP ingredients related to GI and cardiometabolic disorders.

Cardiometabolic and GI disorders often intersect through shared mechanisms, such as low-grade systemic inflammation, oxidative stress, and intestinal dysbiosis [6,7]. Preventive measures focused on lifestyle improvements can cost-effectively mitigate these issues. Studies have reported associations between orange and orange juice consumption and changes in inflammatory markers, oxidative stress, and gut microbiota [27,28]. Similar outcomes have been investigated for upcycled OP ingredients. Orange flavonoids and pectin reach the colon unabsorbed, modulating the gut microbiota to produce beneficial metabolites that help modulat oxidative stress and inflammation modulation [29,30,31]. Figure 3 summarizes the main health effects described for upcycled OP ingredients. Although polyphenols, pectin, carotenoids and EO from OP share many similar health benefits, polyphenols stand out for their particularly strong antioxidant and anti-inflammatory properties. Pectin, as a prebiotic, is better known for its role in regulating glucose, insulin, and lipid levels, as well as promoting a balanced gut microbiota. While EO and carotenoids also provide antioxidant, antimicrobial, and anti-inflammatory effects, evidence of their cardiometabolic and gut benefits is limited, possibly due to their lipophilic nature, which may affect bioavailability and efficacy in cardiometabolic applications. Table 1 outlines the most relevant in vitro and animal studies (preclinical) of upcycled OP ingredients on various GI and cardiometabolic disorders.

#### 3.2.1. Cardiometabolic Health

The global rise in cardiometabolic diseases has led to significant research on dietary interventions that may mitigate these risks. Epidemiological studies reported an inverse relationship between flavonoid and dietary fiber intake and cardiometabolic risk, highlighting their protective roles [32,33]. Upcycled OP ingredients have shown promise in improving markers of cardiometabolic health through diverse mechanisms (Table 1). Flavonoids, particularly hesperidin and PMFs, mitigate oxidative stress, reduce inflammation, and improve lipid metabolism, benefiting cardiovascular health. The endothelium regulates vascular tone, blood flow, and inflammatory and oxidative processes in a healthy vascular system. Endothelial dysfunction disrupts these mechanisms, leading to hypertension and atherosclerosis. On the other hand, these conditions exacerbate endothelial dysfunction, creating a harmful cycle that increases cardiovascular and metabolic risks [34]. The OP-derived flavonoid extracts promote nitric oxide (NO) production, essential for maintaining vascular tone and reducing hypertension and atherosclerosis risk. Hesperidin and PMFs inhibit the expression of endothelin-1 (Edn1) expression while enhancing endothelial NO synthase (eNOS) activity [35,36].

Preclinical studies with PMF-rich extracts demonstrated dose-dependent reductions in systolic and diastolic blood pressure in hypertensive rats, along with decreased oxidative stress markers like malondialdehyde (MDA) and increased antioxidant enzyme activities [36]. These findings highlight upcycled OP ingredients as natural alternatives to conventional vasodilatory agents. While studies on EO, carotenoids, and pectin’s cardioprotective effects are limited, Oboh et al. [37] reported that EO rich in D-limonene has ACE (angiotensin-converting enzyme) inhibitory activity, contributing to blood pressure regulation. Although EO’s ACE inhibition is less potent than lisinopril, its natural origin and lower side-effect profile make it attractive for managing hypertension. Together, these actions reduce vascular inflammation and oxidative stress, breaking the cycle of endothelial dysfunction and hypertension.

Atherosclerosis, a leading cause of stroke and cardiovascular disease, involves lipid abnormalities, oxidative stress and chronic inflammation [38]. OP-derived flavonoids, particularly hesperidin and nobiletin, exhibit anti-atherogenic properties by modulating lipid profiles and suppressing plaque formation. Preclinical studies showed hesperidin-rich extracts reduce adhesion molecules like VCAM-1 and ICAM-1, which mediate endothelial function and plaque deposition [35]. Flavonoids also inhibit platelet-activating factor acetyl-hydrolase (PAF-AH), preventing plaque deposition and vascular damage [38]. Pectin reduces galectin-3 (Gal-3) levels, mitigating inflammation and supporting endothelial function [39]. PMFs extract, rich in sinensetin and nobiletin, suppress trimethylamine (TMA) and trimethylamine-N-oxide (TMAO) production by inhibiting microbial enzymes like cutC/D [40]. As TMAO promotes atherosclerosis, lipid dysregulation, and endothelial dysfunction, modulating the gut microbiota is a promising strategy for reducing cardiovascular risk. Interventions with BCs that reach the colon can selectively suppress TMA-producing bacteria, decrease TMAO levels, and improve vascular health.

OP-derived flavonoids suppress pro-inflammatory cytokines such as TNF-α and IL-6, and PMFs inhibit NF-κB activation, while reducing leukocyte adhesion—an early step in plaque formation [35]. Their antioxidant properties upregulate enzymes such as SOD and catalase and protect endothelial cells from oxidative stress-induced apoptosis. The dual role of flavonoid extracts and pectin in improving lipid profiles and reducing oxidative stress underscores their potential as natural interventions for cardiometabolic health.

Obesity, a condition characterized by an energy imbalance, is a major driver of cardiometabolic diseases, including type 2 diabetes. Its associated complications, such as systemic inflammation and oxidative stress, exacerbate metabolic dysfunction [41,42]. The United Nations’ 2030 Sustainable Development Goals (SDG 3.4) aim to reduce deaths from non-communicable diseases, including obesity, by one-third through prevention and treatment strategies. Traditional obesity management strategies, such as restrictive diets and pharmacological treatments, often yield limited long-term success and adverse effects, driving interest in natural interventions or nutraceuticals [41] like upcycled OP ingredients.

Different categories of upcycled OP ingredients exert anti-obesity effects by modulating lipid metabolism, suppressing adipogenesis, reducing inflammation, and influencing gut microbiota (Table 1). EO and flavonoid-rich extracts regulate lipid profiles, reducing body weight gain, cholesterol, and LDL while increasing HDL. They also enhance antioxidant enzyme activity, reducing oxidative damage [43]. On the other hand, they prevent inflammation by suppressing cytokines like leptin and insulin while inhibiting NF-κB activation, reducing chronic inflammation [44,45,46]. The anti-inflammatory effects of these BCs are complemented by their strong antioxidant properties, which mitigate oxidative stress in adipose and hepatic tissues, further supporting metabolic health. For instance, EO microcapsules reduced fat accumulation in rats fed a high-fat diet by suppressing lipogenesis and improving gut barrier function, thereby mitigating systemic endotoxemia. In parallel, polyphenol-rich extracts reduce adipocyte lipid accumulation by downregulating PPARγ and C/EBPs, which are critical in developing obesity [47]. Specifically, flavonoids from OP, namely naringenin, hesperidin, and nobiletin, modulate PPARγ expression in preadipocytes, suppressing adipogenesis and reducing intracellular lipid accumulation. In parallel, these BCs upregulate adiponectin expression—an adipokine with anti-inflammatory and insulin-sensitizing properties that is typically reduced in obese individuals, thereby contributing to improved metabolic homeostasis. Additionally, obesity-related conditions like non-alcoholic fatty liver disease (NAFLD) benefit from OP ingredients, which decrease liver damage markers and necrosis [48] and reduce high-fat diet-induced pro-inflammatory hepatic cytokines, including IL-6, MCP-1, COX-2, and TNF-α. However, more clinical trials are necessary to confirm the hepatoprotective effects.

Although pectin has demonstrated benefits in improving lipid metabolism and glycemic control in other contexts [49], there is a notable gap in research on OP-derived pectin and its anti-obesity effects. Studies investigating its role in adiposity, fat distribution, or energy metabolism are lacking. Given its known prebiotic properties and potential to modulate gut microbiota, pectin represents a promising candidate for further research into its role in managing obesity through gut microbiota modulation. Exploring these mechanisms may reveal novel applications for this OP-derived compound in reducing body fat and improving metabolic health. The gut microbiota is increasingly recognized as a critical mediator in obesity and metabolic health [50]. Upcycled OP ingredients, particularly polyphenols and pectin, act as prebiotic-like compounds that selectively promote the growth of beneficial bacterial genera, such as *Bifidobacterium* and *Lactobacillus.* These changes in microbial composition enhance the production of SCFAs, such as acetate, propionate, and butyrate, thereby improving gut barrier function, reducing systemic inflammation, and regulating lipid and glucose metabolism [51]. Further, EO supports gut health by increasing microbial diversity, promoting anti-inflammatory bacteria such as *Allobaculum* and *Lactobacillus*, and suppressing pro-inflammatory genera such as *Helicobacter*. These microbiota-mediated effects contribute to improved metabolic profiles, reduced fat storage, and enhanced intestinal integrity [52].

Type 2 diabetes is closely linked to obesity, which triggers inflammation, insulin resistance, and disrupted glucose metabolism [53]. Obesity also alters lipid profiles and induces oxidative stress, worsening diabetes complications. Targeting these shared metabolic pathways, nutraceuticals from OP upcycling can improve lipid metabolism, reduce inflammation, and support gut health, offering a promising approach to prevent and manage diabesity. Diabetes is a multifaceted metabolic disorder characterized by chronic hyperglycemia, insulin resistance, and impaired pancreatic β-cell function [54]. Despite the widespread use of pharmacological treatments, these therapies’ side effects, cost, and limited long-term efficacy have fueled interest in natural alternatives. Upcycled OP ingredients have shown potential as antidiabetic innovative interventions (Table 1). One of the primary mechanisms by which different upcycled OP ingredients exert antidiabetic effects is the regulation of glucose metabolism and postprandial glycemic control. Polyphenols such as hesperidin and narirutin have demonstrated significant inhibitory activity against α-glucosidase and α-amylase enzymes, which are essential for the breakdown of dietary carbohydrates. In vitro studies have shown that these BCs to slow glucose absorption, thereby reducing postprandial blood sugar spikes [53]. EO, particularly those rich in D-limonene, have also exhibited comparable enzyme-inhibitory properties, further supporting their role in controlling postprandial glycemia. These in vitro findings are substantiated by animal studies, in which supplementation with OP-derived polyphenols resulted in reductions in fasting blood glucose of up to 56% and in plasma insulin levels of approximately 32.7%. Mechanistically, these effects are attributed to enhanced insulin signaling pathways, including the upregulation of peroxisome proliferator-activated receptor gamma (PPARγ), glucose transporter type 4 (GLUT4), and insulin receptor expression in adipose tissue [43]. Improvements in hepatic glucose metabolism were also observed, showing increased liver glycogen storage and decreased activity of enzymes such as glucose-6-phosphatase, indicating better glucose utilization.

Beyond glucose metabolism, OP-derived compounds enhance insulin sensitivity and protect the pancreas, two critical factors in managing diabetes. Pectin has been shown to improve insulin sensitivity by modulating the phosphatidylinositol-3-kinase (PI3K)/Akt signaling pathway. In diabetic animal models, pectin supplementation increased Akt phosphorylation and reduced glycogen synthase kinase-3β (GSK3β) expression, enhancing glucose uptake and insulin receptor sensitivity [55]. These metabolic benefits were complemented by improvements in lipid profiles, including reductions in triglycerides, total cholesterol, and LDL cholesterol, along with increased HDL cholesterol levels. Additionally, OP polyphenols have demonstrated protective effects on pancreatic β-cells, which are vital for insulin production. Preclinical studies revealed that upcycled OP ingredients restore pancreatic β-cell architecture, increase insulin-positive cell density, and upregulate antioxidant defenses, including glutathione peroxidase and superoxide dismutase. These effects mitigate oxidative stress and inflammation in pancreatic tissue, delaying β-cell dysfunction and improving overall glycemic control [43].

The anti-inflammatory and antioxidant properties of OP compounds further enhance their anti-diabetic potential. Polyphenols suppress cytokines like TNF-α and IL-6 and inhibit NF-κB activation, while pectin supports SCFA production, improving intestinal integrity and reducing systemic inflammation. Future studies must validate these preclinical results in human populations, optimize dosing strategies, and explore synergistic effects among different OP bioactivities.

#### 3.2.2. Gastrointestinal Health

Emerging evidence reported that upcycled OP ingredients, including polyphenol extracts, EO, and pectin, support GI health by reducing inflammation and oxidative stress, modulating gut microbiota, and potentially contributing to cancer prevention (Table 1). However, a notable gap in the literature is the absence of studies specifically evaluating carotenoids derived from OP in the context of GI and cardiometabolic health. Although carotenoids are recognized BCs present in orange by-products, current research on upcycled OP ingredients has largely focused on polyphenols, EO, and pectin. Consequently, the potential role of OP-derived carotenoids in GI and cardiometabolic health remains largely unexplored and warrants further investigation. The limited number of studies investigating carotenoids derived from OP appears to be multifactorial. From a compositional perspective, although OP contains carotenoids such as β-carotene and lutein, their concentrations are generally lower than those reported in other agro-industrial by-products, including tomato pomace and carrot residues, which are widely recognized as primary carotenoid sources. While direct quantitative comparisons across studies remain limited due to differences in analytical methods and reporting units, the overall trend consistently indicates a lower carotenoid yield in OP matrices. In addition, technological constraints may further limit their exploration. Carotenoids are highly susceptible to degradation induced by light, oxygen, and heat, and their lipophilic nature poses challenges for extraction, stabilization, and incorporation into food systems, particularly when compared to more stable and hydrophilic compounds such as polyphenols and pectin. Finally, current valorization strategies of OP have predominantly focused on polyphenols, EO, and pectin, reflecting both their higher abundance and technological feasibility, which may have contributed to a relative research underrepresentation of carotenoids.

Upcycled OP ingredients significantly contribute to GI health through their anti-inflammatory properties. Chronic inflammation plays a key role in GI disorders like IBD, which causes intestinal mucosal damage, impaired barrier function, and intense immune responses. For instance, hesperidin, nobiletin, and narirutin inhibit inflammatory pathways by reducing pro-inflammatory cytokines, such as tumor necrosis factor-alpha (TNF-α), interleukin-6 (IL-6), and interleukin-1β (IL-1β). Additionally, they downregulate the nuclear factor-kappa B (NF-κB) pathway, a critical mediator of inflammation. Studies in mice with induced colitis demonstrated that OP polyphenol-rich extracts mitigate ulcerative colitis symptoms by decreasing inflammatory markers and preserving colon tissue structure [56]. While EO, particularly D-limonene, further enhances anti-inflammatory effects by reducing intestinal inflammation scores and serum TNF-α levels, comparable to conventional anti-inflammatory drugs like ibuprofen [57]. Pectin also attenuates colitis symptoms by reducing colonic pro-inflammatory cytokines and enhancing fecal SCFA production, which supports intestinal integrity and immune modulation. In addition to their anti-inflammatory effects, the extracts exhibit prebiotic properties that promote gut microbiota diversity and function. The gut microbiota is critical for maintaining intestinal health, and its dysbiosis is implicated in various GI disorders, including IBS, IBD, and colorectal cancer [24,58].

**Figure 4:**
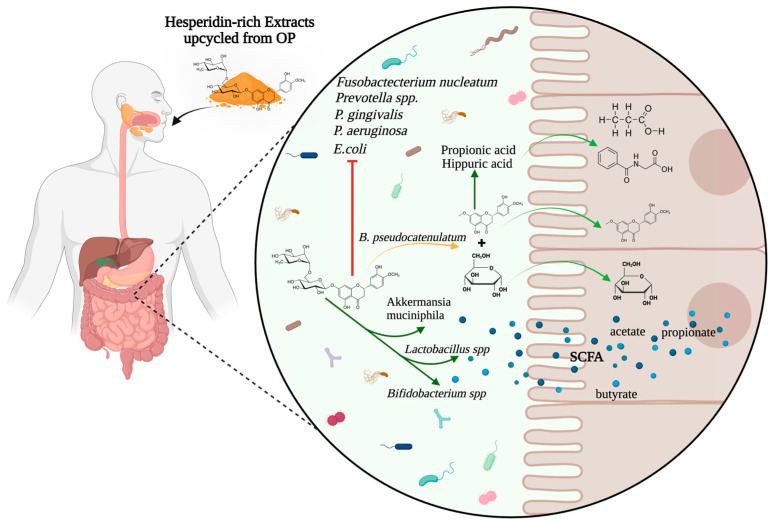
The modulation of gut microbiota by hesperidin-rich extracts and its impact on bacteria groups and modulation of short-chain fatty acid production.

Polyphenols and pectin from OP act as substrates for beneficial gut bacteria, selectively promoting the growth of genera such as *Bifidobacterium* and *Lactobacillus* while reducing harmful bacteria like *Helicobacter* [10,59]. Hesperidin, the major flavonoid present in OP, can modulate gut microbial composition and function, which affects the release of microbial-derived metabolites such as SCFA, sugars, and phenolic acids (Figure 4). Flavonols are active inhibitors of some Gram-negative bacteria, such as *Prevotella* spp., *Porphyromonas gingivalis*, *Fusobacterium nucleatum*, *E. coli*, *Pseudomonas aeruginosa*, and *Clostridium* spp [59]. In addition, hesperidin and other flavonols also inhibit the growth of some Gram-positive bacteria, such as Staphylococcus aureus and Lactobacillus acidophilus. Growing evidence reports associations between polyphenol intake and changes in gut microbiota composition and activity, including increased production of SCFAs in the large intestine [10]. The immunomodulatory actions of hesperidin on the gut and reinforce its role as a prebiotic; however, deeper studies of hesperidin effects on gut microbiota are necessary to completely understand these potential discrepancies.

On the other hand, EO supplementation also increased gut microbial diversity, elevated beneficial bacteria, and reduced pro-inflammatory taxa, thereby improving gut barrier function and reducing systemic endotoxemia. Prebiotic effects are also linked to enhanced SCFA production, including acetate, propionate, and butyrate, which strengthen the intestinal barrier, reduce inflammation, and regulate gut motility [44,45,52]. These findings underline the role of upcycled OP ingredients in restoring microbial balance and supporting GI health.

The antimicrobial properties of OP-derived compounds provide additional protection for GI health. Disruptions in the gut microbial ecosystem often leads to infections and exacerbation of GI conditions. Phenolic compounds and EO exhibit strong antibacterial activity against pathogens such as *Helicobacter pylori*, *Escherichia coli*, and *Listeria monocytogenes*, which are commonly linked to GI infections and complications. For instance, D-limonene-rich EO have been shown to inhibit the growth of *Helicobacter pylori*, a bacterium strongly associated with gastritis, ulcers, and gastric cancer. Polyphenols suppress bacterial growth and reduce biofilm formation, enhancing their antimicrobial efficacy. These effects contribute to maintaining a balanced gut microbiome and preventing infection-driven GI disorders.

The antineoplastic potential of BCs from OP adds another dimension to their contributions to GI health, particularly in the context of colorectal cancer, which is the third most common cancer worldwide. Chronic inflammation and oxidative stress are key drivers of neoplastic transformations, and OP polyphenols-rich extracts counteract these mechanisms through their antioxidant and apoptosis-inducing properties. EOs have demonstrated cytotoxic effects on colorectal cancer cells by disrupting mitochondrial membrane potential and promoting apoptosis through caspase activation pathways. Similarly, polyphenol-rich extracts inhibit colon cancer cell proliferation by arresting the cell cycle in critical phases and reducing cancer stem cell markers such as PROM1 and LGR5. These effects are complemented by increased glutathione levels and antioxidant enzyme activities, which protect against DNA damage and oxidative stress-induced carcinogenesis. Pectin further contributes to cancer prevention by modulating gut microbiota composition and reducing inflammatory mediators that are associated with tumor progression.

In summary, upcycled OP ingredients exhibit a broad spectrum of benefits for GI health by addressing key aspects of inflammation, microbial balance, neoplastic prevention, and antimicrobial defense. Their multifaceted actions not only mitigate symptoms of GI disorders such as IBD and IBS but also provide a preventive approach to conditions like colorectal cancer.

#### 3.2.3. Clinical Evidence: Current Status and Research Gaps

Based on the search, fourteen completed clinical trials with results published in peer-reviewed journals were identified ton ClinicalTrials.gov and Scopus. All trials presented in Table 2 were registered, conducted, and published, and were selected based on predefined eligibility criteria. Only trials investigating ingredients derived exclusively from OP and reporting outcomes related to GI or cardiometabolic health were included; registered-only or ongoing trials without published results were excluded. Most clinical trials focus on GI and cardiometabolic health, accounting for 55% of the identified studies. The studies used various methodologies, including randomized, double-blind, and placebo-controlled designs, involving both healthy individuals and those with mild cardiometabolic risks. Polyphenol-rich and fiber-rich extracts are among the bioactive compounds most frequently investigated. Reported outcomes include changes in gut health parameters, glycemic response, and cardiovascular markers. Polyphenol-rich and fiber-rich extracts are among the bioactive compounds most frequently investigated in clinical trials.

Because of its high fiber content, OP powder is primarily analyzed for its effects on the gut microbiota and postprandial glucose response. A relevant study by Dennis-Wall et al. [60] found that OP supplementation significantly increased weekly stool frequency and altered microbial populations, particularly *Lachnospiraceae* and *Ruminococcaceae*, which are associated with gut health, although participants reported GI symptoms, including gas and bloating, likely due to fiber fermentation in the colon. In contrast, Alexander et al. [61] found no significant changes in bowel habits or microbiota diversity among healthy adults consuming OP powder daily, suggesting that its impact may be minimal in individuals with a balanced gut microbiota. These contrasting results underline the importance of individual variability and the need for further research in diverse populations. In addition, hesperidin-rich extracts have demonstrated promising prebiotic effects. Recently, Maurer Sost et al. [62] observed a significant shift in SCFA profiles and reductions in inflammatory markers, such as fecal calprotectin, indicating improved gut health. However, consistent changes in microbiota diversity and functionality still need to be clarified, aligning with the findings of Alexander et al. [61]. Applying advanced metagenomic and metabolomic techniques in future studies could provide a more detailed understanding of microbiota-mediated mechanisms and clarify these inconsistencies.

Multiple clinical trials have demonstrated that OP interventions can modulate postprandial glucose levels and insulin responses. Studies from Chen et al. and Guzman et al. [63,64] reported significant reductions in postprandial glucose levels and delayed insulin peaks following OP supplementation, particularly at higher doses. These effects are attributed to the high fiber content of OP, which slows digestion and glucose absorption, and are particularly relevant for individuals with elevated metabolic risks. Regarding cardiometabolic outcomes, Papagianni et al. [65], found that a functional meal enriched with OP extract reduced LDL cholesterol levels shortly after intake, indicating that OP-derived polyphenols may significantly acutely modulate lipid profiles. Similarly, Salden et al. [66], demonstrated that hesperidin supplementation in overweight individuals reduced systemic inflammation markers and systolic and diastolic blood pressure. These findings underscore the potential of OP-derived polyphenols to improve vascular function and decrease cardiovascular risk factors over time.

Despite these promising results, several critical research gaps remain. Notably, no clinical trials have evaluated the health impacts of OP-derived pectin, despite the strong effects demonstrated in in vitro and animal models for gut microbiota modulation, glucose metabolism, and lipid regulation. This lack of clinical evidence for pectin and carotenoids represents a missed opportunity to explore the full range of OP bioactivities. Additionally, while OP-derived flavonoids have shown antineoplastic potential in preclinical models, their effects on cancer risk or progression remain unvalidated in human studies.

**Table 1:** Compilation of cardiometabolic and gastrointestinal health effects of upcycled OP ingredients evaluated by in vitro and animal studies.

Bioactivity	Ingredient	Ingredient Composition	Model	Main Outcomes	Ref.
**Antihypertensive**	EO	D-limonene (92.14%), b-myrcene (2.7%)	In vitro (Enzymatic assay)	ACE inhibition IC_50_ = 31.79 µg/mL (lisinopril, IC_50_ = 24.03 µg/mL).	[37]
	PMFs-rich oil	D-limonene (80.9%), Nobiletin (47.72 mg/L), tangeretin (25.55 mg/L), sinensetin (5.10 mg/L)	Animal (N^ω^-nitro-L-arginine-induced hypertensive rat model)	↓ both systolic and diastolic pressure in hypertensive rats, with a dose-dependent effect ↑ NO content in serum, heart, liver and kidney compared to untreated hypertensive rats ↓ Malondialdehyde levels, indicating lower oxidative stress ↓ Endothelin-1 (a vasoconstrictor) and ↑ CGRP (a vasodilator) levels in serum ↑ eNOS and nNOS expression ⊣ iNOS, similar to captopril.	[36]
	Fiber-rich extract	ND	In vitro (Enzymatic assay)	↑ ACE-inhibitory activity (1% extract reach 58.5% ACE inhibition) ↑ Antioxidant activity compared to the control	[67]
	Purified flavonoids fraction	hesperidin, linarin, isorhoifolin, and diosmetin,	Animal (Chronic venous hypertension)	↓ leukocyte-endothelium interactions ↓ the increase in venular diameter (observed in untreated animals) ↑ blood flow and capillary function	[68]
**Vasoprotective/** **Anti-atherogenic**	Polyphenols-rich extract	Hesperidin (6 mg/g), narirutin (0.5 mg/g), phenolic acids (≈0.2 mg/g)	In vitro (Human dermal microvascular endothelium cells—HMEC-1cells line)	⊣ TNF-α-induced inflammation by ↓ expressing inflammatory markers like VCAM-1, ICAM-1, and Il1β. ↓ gene expression of endothelin-1, a vasoconstrictor, indicating potential vascular protective effects against endothelial dysfunction ↓ levels of bile acids such as choline and deoxycholic acid	[35]
	PMFs extract	Nobiletin, tangeretin, sinensetin	In vitro (Enzymatic assay)	⊣ activities of the cntA/B and cutC/D enzymes (critical in converting dietary components in TMA in the gut microbiome, which subsequently elevates TMAO levels) ↓ in TMAO formation by suppressing the expression of the FMO3 gene (responsible for converting TMA into TMAO in the liver) in hepatocytes	[40]
**Anti-obesogenic and anti-lipidemic**	OP powder	ND	Animal (Male Wistar albino rats)	↓ body weight gain in rats compared to commercial-fed group ↓ glycemic index (»31.5%) ↑ HDL and ↓ LDL cholesterol levels ↑ antioxidant enzyme activities (catalase, superoxide dismutase, and glutathione S-transferase) in the liver and heart tissues of rats	[69]
	EO microcapsules	D-limonene (97.42%)	Animal (Male SD rats with high-fat diet)	↓ body weight gain by up to 70% and fat accumulation by 33.8% in obese rats, even with a high-fat diet. ↓ total cholesterol and LDL-cholesterol levels ↓ endotoxin levels in the blood of obese rats by 8.8%, which is associated with lower inflammation and improved gut barrier function. ↓ leptin, neuropeptide and insulin levels ↓ adipocyte size	[44,45]
	Polyphenols-rich extract	-	Animal (High-fat diet-fed, streptozotocin-induced diabetic rat model)	↓ weight gain ↓ levels of triglycerides, total cholesterol, and LDL cholesterol ↑ HDL cholesterol = adipose tissue structure in rats, with reductions in cell enlargement and better distribution of fat cells.	[43]
	Polyphenols-rich extract (after digestion)	Hesperidin (3.59 mg/g), narirutin (0.63 mg/g)	In vitro (Murine fibroblast cell line—3T3-L1 cells)	↓ 13.6% in lipid accumulation was observed in adipocytes treated with 150 µg/mL extract Ø antilipolytic action	[47]
	PMFs-rich extract	ND	Animal (Zucker Diabetic Fatty rats)	↑ free fatty acids, cholesterol, and LDL levels = body weight or BMI ⊣ inflammation in adipose tissue, reducing markers such COX-2, ICAM-1, and TNF-α	[46]
	Polyphenols-rich extract	ND	Animal (Type 2 diabetes rats induced by nicotineamide and streptozotocin)	↓ total cholesterol, triglycerides, LDL, and free fatty acids and ↑ HDL cholesterol ↓ lipid peroxidation levels and ↑ antioxidant markers like glutathione and enzyme activities (glutathione peroxidase and glutathione-S-transferase) ↑ mRNA expressions of adiponectin in adipose tissue	[70]
**Antidiabetic**	Polyphenols-rich extract	ND	Animal (Type 2 diabetes rats induced by nicotineamide and streptozotocin)	↓ glucose, insulin and C-peptide levels ↑ liver glycogen content and ↓ liver enzyme activities (glucose-6-phosphatase and glycogen phosphorylase) ↑ mRNA expressions of insulin receptor β-subunit and GLUT4	[70]
	PMFs-rich extract	ND	Animal (Zucker Diabetic Fatty rats)	↓ fasting blood glucose levels, though less effectively than metformin (positive control). Ø glucose tolerance ⊣ inflammation in adipose tissue, reducing markers like COX-2, ICAM-1, and TNF-α, which are linked to inflammation in diabetes.	[46]
	Polyphenols-rich extract	Hesperidin (55.59 mg/g DW), narirutin (31.45 mg/g DW), nobiletin (37.05 mg/g DW), sinensetin (67.26 mg/g DW)	In vitro (Enzymatic inhibition assay)	⊣ α-glucosidade (IC_50_ = 16.25 µg/mL) ⊣ glycation activity (IC_50_ = 93.26 µg/mL)	[53]
		ND	Animal (High-fat diet-fed and streptozotocin-induced diabetic rat model)	↓ fasting blood glucose levels (by approximately 56%) and plasma insulin levels (by 22.9% to 32.7%) ↑ expression of insulin-signaling molecules (PPARγ, GLUT4, and insulin receptor) in adipose tissue restored pancreatic β-cell architecture ↑ intensity of insulin-positive cells in the pancreatic islets	[43]
	Pectin	Mw 3.063 × 10^5^ Da; DE (≈70), GalA (»70%)	Animal (SD rats and diabetes induced by streptozotocin)	↓ fasting blood glucose levels and improve glucose and insulin tolerance ↑ p-Akt and ↓ of GSK3β expression in the PI3K/Akt pathway (beneficial for insulin sensitivity)	[55]
**Hepatoprotective**	OP powder	Hesperidin, hesperitin, nobiletin, tangeretin	Animal (Liver injury induced by CCl_4_)	↓ levels of liver enzymes AST and ALT, markers of liver damage at doses of 10 and 100 mg/kg ↑ SOD and GPx (mitigate oxidative stress) ↓ lipid peroxidation, evidenced by lower TBARS ⊣ necrosis and other structural liver damage	[48]
	EO (by sniffing)	ND	Animal (NAFLD induced by a high-fat diet)	↓ lipid accumulation in liver cells ↓ levels of triglycerides, total cholesterol and LDL cholesterol ↓ expression levels of acetyl–CoA carboxylase and CYP2E1 ↑ expression levels of PPAR-α and CPT-1	[71]
	OP powder	Pectin (19.3 mg/g DW), EO (0.20) (%), Narirutin (1.11 mg/g DW), Hesperidin (0.24 mg/g DW)	Animal (NAFLD, lipid metabolism disorders, and gut microbiota dysbiosis induced by high-fat diet in SD rats)	↓ weight gain during high-fat fed ↓ hepatic fat accumulation caused by a high-fat diet ↓ serum levels of total cholesterol, triglycerides, LDL cholesterol, and alanine aminotransferase ↓ release of pro-inflammatory cytokines (IL-6, MCP-1, TNF-a, COX-2) compared to control group (*p* < 0.05)	[72]
	Flavonoids-rich extract	Naringenin, diosmin, quercetin, hesperidin, naringin, rutin	Animal (Liver injury induced by paracetamol in Wistar rats)	↓ levels of liver enzymes (ALT, AST, ALP, LDH, GGT) and total bilirubin ↑ levels of liver GSH and the activities of antioxidant enzymes (SOD, GPx, GST) ↓ inflammation by decreasing serum levels of TNF-α and increasing IL-4 ↓ expression of proapoptotic markers (p53, Bax, and caspase-3) ↑ Bcl-2 (antiapoptotic) levels	[73]
**Antineoplastic**	EO	D-limonene (88.07%)	In vitro (Human colorectal carcinoma and hepatocellular carcinoma cells, HTC116 and HepG2 cell lines, respectively)	⊣ proliferation of colon cancer HCT116 (IC50 = 0.35 μL/mL) ⊣ proliferation of HepG2 hepatoma cells (IC50 = 0.29 μL/mL)	[74]
	Polyphenols-rich extract	TPC (2.83 mg/g DW), TFC (2.143 mg/g DW)	In vitro (Esophageal cancer cells- YM1 cells line)	↓ the growth of esophageal cancer stem cells ↓ late-stage apoptosis ⊣ cancer cells from completing their growth cycle, specifically stops S-phase ↓ oxidative stress markers by lowering MDA, a marker of lipid peroxidation ↑ antioxidant enzyme levels (SOD and total antioxidant capacity)	[75]
		Rutin (12.53 mg/g DW), naringin (10.67 mg/g DW), quercitrin (8.91 mg/g DW)	In vitro (Metastatic human colorectal cells)	⊣ Metalloproteinase activity in colon cancer cells in a concentration-dependent manner. ↑ glutathione reductase and glutathione peroxidase are beneficial in treatment of early-stage colon cancer but might not be beneficial in managing late-stage colon cancer.	[76]
		ND	In vitro (3D cell model of colorectal cancer)	⊣ cell proliferation in a dose- and time-dependent manner and colony formation (a marker of self-renewal) ↓ cancer stem cell markers (PROM1, LGR5) and ALDH+ cell population	[77]
		Hesperidin (5.65 mg/mL), tangeretin (11.65 mg/mL), sinensetin (13.86 mg/mL), nobiletin (5.32 mg/mL)	In vitro (Hepatocellular carcinoma cells—HepG2 cell lines)	↓ Cell viability decreases in a dose-dependent manner, linked to G0/G1 phase arrest and enhanced apoptosis (programmed cell death). Extract disrupts mitochondrial membrane potential, leading to the release of pro-apoptotic proteins ↑ Bax/Bcl-2 ratio, a critical marker of cell apoptosis, thus promoting cell death.	[78]
			Animal (Xenograft model in male nude mice)	⊣ tumour growth without causing toxicity, as evidenced by the stable body weight of treated animals. The highest dose (10 mg/kg) reduced tumour size by approximately 73% compared to untreated controls	
	PMFs-rich extract	98% of nobiletin, tangeretin and 5-Demethylnobiletin	In vitro (Human gastric adenocarcinoms)	⊣ cell proliferation →^+^ apoptosis in the cancer cells. The ↑ expression of apoptosis-related proteins like Caspase3, Caspase9, and PARP1 supported this. →^+^ RARβ, a mechanism that induces apoptosis in gastric cancer cells.	[79]
		Nobiletin (92.8 mg/g DW), sinensetin (70.5 mg/g DW), tangeretin (9.8 mg/g DW)	In vitro (3D cell model of colorectal cancer)	⊣ cell proliferation →^+^ cell cycle arrest (G2/M phase)—a critical point for controlling cell division, which can prevent cancer cell proliferation ↑ apoptosis (programmed cell death), especially those collected at earlier stages of culture ↓ ALDH^+^ population	[80]
**Anti-colitic**	OP powder	Total Fibre 67.42% (insoluble 93 and soluble 7%); TPC (211 mg GAE/100 g)	Animal (Colitis induced by DSS in mice)	↓ iNOS expression compared to the DSS control group ↓ expression of inflammatory cytokine (TNF-α, IL-1β, IL-6) and adhesion molecules (ICAM-1) ↑ expression of intestinal barrier proteins (MUC-3, Occludin, and ZO-1) ⊣ weight loss compared to the control group ↓ disease activity index and colonic weight-to-length, indicating a less severe inflammatory response compared to the DSS control group	[81]
	D-limonene	ND	In vitro (Mouse embryonic fibroblasts) Animal (rat model of colitis)	⊣ NF-κB Activation ↑ transepithelial electrical resistance ↓ intestinal inflammation scores compared to untreated colitis-induced rats. ↓ TNF-α serum levels ↓ loss of weight and the colon length ↓ severe inflammatory and necrotic damage compared to untreated colitis-induced rats	[57]
	Polyphenols-rich Extract	Hesperidin (14.8), narirutin (6.53), sinensetin (7.08), nobiletin (5.65), ferulic acid (12.4) (mg/g DW)	*Animal* (DSS-induced acute colitis)	↓ typical ulcerative colitis symptoms, such as weight loss, diarrhea, and rectal bleeding. ↓ inflammatory markers such as myeloperoxidase activity, TNF-α, and IL-6 in the colon and serum. ↑ anti-inflammatory cytokine IL-10 ⊣ colon tissue from DSS-induced damage, including structural deterioration and neutrophil infiltration. ⊣ NF-κB pathway activation—a major inflammatory pathway linked to the progression of colitis	[56]
	Pectin	ND	Animal (C57BL/6N mice, TNBS-induced colitis)	↓ colitis symptoms and colonic tissue damage ↓ weight loss, food intake impact, and colon tissue inflammation after colitis induction ↓ of colonic pro-inflammatory cytokines, specifically IL-1β, IL-6, and TNF-α, which are central to colitis pathogenesis. ↑ fecal levels of propionic acid, a SCFA linked to anti-inflammatory effects	[82]
	Dry Flour (insoluble fiber with bound polyphenols)	Fiber (35.2 g/100 g); TPC (22.64 mg GAE/g);	In vitro (Human colorectal cancer cell lines HT-29 and Caco-2, LPS-induced inflammation)	↓ expression of pro-inflammatory cytokines IL-1β and IL-6 ↑ expression of anti-inflammatory cytokines IL-10 and TGFβ ↓ TLR4 protein expression ↓ NLRP3 inflammasome expression ⊣ NF-kB pathway via ↓ p-IkBα expression	[83]
**Gut modulatory/Prebiotic**	OP powder	Pectin (19.3 mg/g DW), EO (0.20) (%), Narirutin (1.11 mg/g DW), Hesperidin (0.24 mg/g DW)	Animal (NAFLD, lipid metabolism disorders, and gut microbiota dysbiosis induced by high-fat diet in SD rats)	Phylum level Changes: ↑ the level of *Firmicutes* while ↓ the level of *Campylobacterota* Genus-level Changes: ↑ *Faecalibaculum* and *Lactobacillus* and ↓ the abundance of *Helicobacter*, *Blautia*, and *Bacteroides* ↑ the abundance of *Lachnospiraceae NK4A136 group* was reported following supplementation	[72]
	EO microcapsules	97.42% D-limonene	Animal (SD rats with high-fat diet)	↑ the relative abundance of *Actinobacteria* and *Bacteroidetes and* ↓ of *Firmicutes phylum* ↑ the relative abundance of *Allobaculum*, *Bifidobacterium* and *Lactobacillus genus* ↑ b-diversity after EO microcapsules intake	[44]
	EO	D-limonene (794.5), linaloo (8.3) (mg/mL)	Animal (Healthy status)	↑ diversity in the gut microbiota of the cecum and colon ↑ relative abundance of *Lactobacillus* and a reduction in *Bacteroides* ↑ *Firmicutes*-to-Bacteroidetes (F/B) ratios	[52]
	Flavonoid-rich extract	Hesperidin (40.16), narirutin (4.57), nobiletin (5.67), sinensetin (1.85) (mg/g DW)	In vitro (Healthy human fecal sample)	No significant changes in diversity and microbial richness Phylum-Level Changes: ↑ *Bacteroidetes*, *Actinobacteria* and *Proteobacteria*. ↓ *Firmicutes*. Genus-level Changes: ↑ *Bifidobacterium*, *Lactobacillus* and *Sutterella* ↑ SCFA production compared to the control.	[83]
		Hesperidin (88.2), naringin (6.5) (%)	In vitro (Model of the colon (TIM-2))	Phylum-Level Changes: ↑ *Bacteroidetes* and ↓ *Firmicutes*. Genus-level Changes: ↑ *Enterococcus* and *Roseburia*, along with *Bacteroides*. ↑ Acetate, propionate and butyrate production for both 250 and 350 mg/day supplementation.	[84]
	Pectin	GalA (74.75), HG (68.72) (%) Mw = 1.88 × 10^5^ g/mol	Animal (Type 2 diabetic mice)	↓ diversity of microorganisms in gut after 28 days consumption ↓ the abundance of *Alistipes*, *Helicobacter* and *Oscillibacter* ↑ the relative abundance of *Dubosiella*, *Akkermansiaceae*, and *Atopobiaceae* ↑ SCFA production (acetate, propionate and butyrate) ↓ 74.35% insulin resistance	[51]
	Polyphenol-rich extract	TPC (29.27 mg GAE/g); TFC (10.54 mg QE/g);	In vitro (Probiotic growth assay—*L. fermentum* NCDC141 and *L. rhamnosus* NCDC347 vs. *E. coli* and *E. faecalis*)	Highest prebiotic activity score compared to pathogens (*E. coli*) ↑ growth of *L. fermentum* and *L. rhamnosus* ↓ growth of *E. coli* and *E. faecalis*	[85]
**Antimicrobial**	Essential Oil	D-limonene (88%)	In vitro (Broth microdilution method)	↓ *Helicobacter pylori* (MIC = 3.90 mg/mL)	[86]
	Polyphenols-rich extract	Hesperidin (27.6), narirutin (4.4), sinensetin (1.1), nobiletin (1.1), tangeretin (0.8) (mg/g DW)	In vitro (Broth microdilution method)	↓ Growth and viability of oral bacterial strains associated with caries: *Streptococcus mutans* (MIC = 13.0; MBC = 37.7 mg/mL and *Lactobacillus casei* (MIC = 20.0 and MBC = 43.3 mg/mL). The combination of 0.1% chlorhexidine and 120 mg/mL extract was more effective as an antibacterial agent than 0.2% Chlorhexidine.	[85]
		Narirutin (19.86), naringin (18.21), hesperitin (11.79) (%)	In vitro (Agar well diffusion method)	The 0.5 mg/mL extract ↓ growth of bacteria known to cause GI infections: *Bacillus cereus*, *Listeria monocytogenes*, *Yersinia enterocolitica* and *Escherichia coli*.	[87]

Abbreviations: OP—orange peels; ND—not defined; EO—essential oils; PMFs—polymethoxyflavones; TPC—total phenolic contente; GAE—gallic acid equivalents; SD—Sprague–Dawley; NAFLD—non-alcoholic fatty liver disease; DSS—dextran sulfate sodium; TNBS—2,4,6-trinitrobenzenesulfonic acid solution; ACE—angiotensin-converting enzyme; NO—nitric oxide; eNOS—endothelial nitric oxide synthase; nNOS—neuronal nitric oxide synthase; iNOS—inducible nitric oxide synthase; TNF-α—tumor necrosis factor; IL—interleucina; TMA—trimethylamine; QE—quercetin equivalent; TMAO—trimethylamine N-oxide; HDL—high-density lipoprotein; LDL—low-density lipoprotein; AST—aspartate aminotransferase; ALT—alanine aminotransferase; SOD—superoxide dismutase; GPx—glutathione peroxidase; TBARS—thiobarbituric acid reactive substances; MDA—malondialdehyde; ALDH—aldehyde dehydrogenase; NF-κB—factor nuclear kappa B; SCFA—short-chain fatty acids. ↑ Increase; ↓ decrease; ⊣ inhibition; = equal; Ø no changes.

A critical appraisal of the available human evidence reveals important methodological heterogeneity that limits direct comparisons across trials. Sample sizes are generally small, ranging from 12 to 111 participants, which reduces statistical power and limits generalizability. Intervention durations vary considerably, from acute single-dose designs to 12-week protocols, making it difficult to draw conclusions about long-term efficacy. Furthermore, the OP ingredients investigated differ substantially in terms of extraction method, standardization, and dose, with hesperidin content ranging from 450 mg/day to undefined amounts in whole pomace preparations. Most trials recruited healthy adults or individuals with mild cardiometabolic risk, restricting translational relevance to clinical populations with chronic disease. Outcome measures are also inconsistent across trials—while some report postprandial glycemic markers, others focus on microbiota composition or lipid profiles—precluding meta-analytic synthesis. Collectively, these factors indicate that the current clinical evidence base, while promising, remains preliminary and insufficient to support definitive health claims for upcycled OP ingredients.

Addressing these gaps will require well-designed, long-term clinical trials in diverse populations, including older adults and individuals with chronic conditions. Future studies should employ advanced analytical tools such as metagenomics and metabolomics to better elucidate microbiota-mediated mechanisms and include comparative arms against conventional treatments or commercial fiber supplements to better position OP ingredients within existing health strategies. Notably, no active clinical trials investigating upcycled OP ingredients are currently registered in ClinicalTrials.gov, underscoring the need for greater investment and collaboration between academia and industry to advance this field.

As sustainability and circular economy frameworks gain increasing attention, OP-based functional ingredients align well with societal trends, offering a unique opportunity to combine health benefits with environmental responsibility. While the existing evidence highlights the potential of upcycled OP ingredients to support gut health, glycemic control, and cardiometabolic health, fully realizing this potential will depend on addressing the identified research gaps through rigorous clinical investigation.

**Table 2:** Main registered Clinical Trials in Clinical.gov involving upcycled OP ingredients as promoters of gastrointestinal and cardiometabolic healthy benefits.

Type of OP-Derived Ingredient	Condition or Health Effect	Clinical Study (Location)	Study Design	Participants	Dose/Intervention	Main Outcomes	Ref.
**Orange pomace**	Gastrointestinal function	NCT02979496 (USA)	Randomized, blinded, placebo-controlled	Healthy adults (62% females), 111 on pomace, 110 control	Pomace beverage 473 mL/day (10 g fiber) for 3-week period	↑ stool frequency (*p* = 0.0281) ↑ GI symptoms (gas and bloating) ↑ mean Bristol Stool Form Scale scores (*p* = 0.04) ↑ *Lachnospiraceae* and *Ruminococcaceae*.	[60]
	Digestive health/gut microbiota	NCT03749031 (Helsinki)	Randomized, double-blinded, crossover, placebo-controlled	91 healthy subjects, aged 18–61 years	Orange juice ~ 470 mL/day (with/without 10 g pomace) for 4-week period	No changes in bowel habits or microbiota α- or β- diversity. At week-4 orange juice with 10 g pomace increases (*p* = 0.066) GI symptoms	[61]
	Glycemic regulation	NCT04369716 (USA)	Randomized, crossover, 3-arm	17 healthy adults aged 20–45 years with a BMI of 20.0–24.9 kg/m^2^, blood glucose < 100 mg/dL	100% orange juice—OJ (250 g); 100% orange juice enzyme-treated juice—OPF (157 g juice with 100 g pomace); raw orange—WOF (227 g edible portion of navel orange) for 3–4 weeks period	Glucose postprandial iAUC was not significantly lower in OPF compared to the OJ or WOF (*p* = 0.57) OPF ↓ the postprandial glucose Cmax compared with OJ (*p* = 0.002)	[64]
		NCT03685201 (USA)		45 healthy adults, 47% female, aged 20–45 years, BMI 20.0–24.9 kg/m^2^, blood glucose < 100 mg/dL		Glucose postprandial iAUC was significantly lower in WOF compared with OPF (*p* = 0.02) and OJ (*p* = 0.001) OPF ↓ the postprandial glucose Cmax compared with OJ (*p* = 0.001)	
		NCT02112851 (USA)	Randomized, placebo controlled, double blind, postprandial crossover, 3-arm	34 overweight men, aged 30–65 years, BMI 25–29.9 kg/m^2^, not diabetic or suffer from other endocrine disorders	240 mL placebo or low-dose/high-dose orange pomace (2.55 g or 5.48 g fiber)	↓ insulin Cmax, delayed glucose response, decreased 2 h post breakfast insulin AUC by 23% to the placebo	[63]
	Acute glycemic response	NCT02962375 (USA)	Randomized, single-center, 2-arm, crossover	12 healthy adults, aged 20–45 years, BMI 20.0–24.9 kg/m^2^, blood glucose < 5.6 mmol/L	Orange juice (~250 mL) with/without 5 g enzyme-treated orange pomace	↓ blood glucose after ingesting juice with pomace (*p* = 0.02), no insulin difference responses	[88]
**Orange Peel Extract (polyphenol-rich extract)**	Cardiometabolic risks	NCT05771571 (Greece)	Randomized, acute, single-blinded, crossover	21 participants with cardiometabolic risk, aged 30–65 years	A fat and carbohydrate meal of mashed potatoes, homogenized with refined olive oil (50 mL) or the functional olive oil with 10% orange peel extract	↓ LDL-cholesterol levels post-meal after functional olive intake (*p* < 0.05)	[65]
**Hesperidin-rich extract**	Cardiovascular health	NCT02228291 (Netherlands)	Randomized, double-blind, placebo-controlled, parallel-group	68 healthy subjects, aged 18–65 years, BMI 25.0–35.0 kg/m^2^, fasting glucose < 7.0 mmol/L, normal hemoglobin A1c (4.4 to 6.2%)	500 mg Cordiart^®^ (450 mg hesperidin) for 6-week period	↓ soluble adhesion molecules (sVCAM-1, sICAM-1) and selectins ↓ systolic and diastolic blood pressure; = flow-mediated dilation (*p* = 0.05)	[66]
	Gut microbiota modulation	NCT02610491 (Germany)	Randomized, double-blind, placebo-controlled, parallel-group	53 adults, aged 18–65 years, at risk for MetS(presenting with 2 out of 5 criteria from diagnostic criteria of metabolic syndrome)	500 mg/day orange extract (>80% hesperidin; Microbiomex^®^) or placebo (cellulose) for 12 week period	Shift in SCFA profile (*p* = 0.022) towards ↑ butyrate ↓ fecal calprotectin levels (*p* = 0.058) ↑ *Roseburia* spp. (*p* = 0.049)	[62]

Abbreviations: USA—United Stated of America; OJ—100% orange juice; OPF—enzyme-treated orange pomace fiber containing orange juice; WOF—whole orange fruit; Cmax—maximal concentration; BMI—body mass index; iAUC—glucose positive incremental area under curve; GI—gastrointestinal; SCFA—short-chain fatty acids. ↑ Increase; ↓ decrease; = equal.

## 4. Commercial Upcycled Orange Peel Ingredients: Market Applications

The upcycling of OP as a sustainable source of BCs has spurred the development of innovative nutraceutical ingredients targeting GI, cardiometabolic, and broader health applications. Several commercial ingredients have emerged, translating preclinical and early clinical findings into real-world applications. Table 3 summarizes those aligned with the scope of this review, focusing on GI and cardiometabolic health outcomes. Inclusion was restricted to ingredients derived exclusively or predominantly (>80%) from OP or other orange by-products and supported by preclinical and/or clinical evidence in the peer-reviewed literature. Most commercially available ingredients are standardized polyphenol-rich extracts, particularly focused on hesperidin content, addressing health areas such as gut health, cardiovascular support, energy metabolism, and weight management. It should be noted that some ingredients discussed below are derived from immature sweet oranges rather than mature OP. Additionally, while the clinical studies have been published in peer-reviewed journals, some were conducted or financially supported by ingredient manufacturers. Although peer review provides an important layer of scientific scrutiny, potential conflicts of interest cannot be entirely excluded, and findings should therefore be interpreted with appropriate caution.

Regarding gut health, MicrobiomeX^TM^ (Solabia Nutrition) is a patented extract from immature oranges and grapefruit (94:6%), standardized to contain more than 80% hesperidin and 5% naringin. In vitro, ex vivo, and human clinical evidence supports its ability to modulate gut microbiota composition, increase butyrate production, and reduce markers of gut inflammation [62,84,89]. Building on this evidence, Lepivits^TM^ incorporated MicrobiomeX^TM^ into two gut health supplements with distinct therapeutic targets. Candovits^TM^ combines 400 mg of MicrobiomeX^TM^ with oregano extract, propolis, olive leaf extract, and garlic, targeting intestinal microbial balance and natural defense reinforcement, particularly in individuals experiencing microbial imbalances such as candidiasis. Permeavits^TM^ combines 500 mg MicrobiomeX^TM^ with prickly pear extract, olive polyphenols-extract, and bromelain, targeting digestive mucosal integrity and addressing gastrointestinal discomforts such as bloating and reflux. These multi-ingredient formulations illustrate how a clinically validated OP-derived ingredient can serve as an evidence-based core component in differentiated nutraceutical products; however, the effects observed in these end-user formulations cannot be attributed exclusively to the MicrobiomeX^TM^.

In the cardiovascular domain, Cordiart^TM^ is a water extract from immature sweet oranges, grown in the Mediterranean region, validated in a RCT (*n* = 68, 500 mg/day, 6 weeks), demonstrating improvements in arterial flexibility, blood pressure, and a significant reduction in endothelial adhesion molecules [66]. Building on these data, a new ingredient, Actiful^TM^, was launched in 2022, combining immature sweet orange extract (>65% hesperidin) with pomegranate extract to support healthy aging and longevity synergistically. Clinical study (NCT03781999) conducted with senior subjects 60+ years and published in international peer-reviewed journals [90,91], demonstrated that Actiful^TM^ supplementation improved well-being, reduced oxidative stress, and enhanced markers of cellular longevity after four weeks of supplementation. Cardiose^TM^ (HTBA) is similarly positioned for cardiovascular health and sports performance; however, available clinical evidence derives exclusively from studies in amateur cyclists (NCT04597983) [92,93], and further trials in broader populations are needed to substantiate cardiovascular health claims. Recently, LifeExtension^TM^ incorporated Cardiose^TM^ (500 mg) into NitroVascTM, marketing it with similar health claims; however, the evidence remains based solely on clinical data from amateur cyclists rather than new clinical trials. WATTS’UP^TM^ (Solabia Nutrition), a 2S-hesperidin-rich extract from immature sweet oranges. Although commercially positioned for sports performance enhancement, the available clinical evidence primarily supports cardiovascular outcomes, including improvements in aerobic and anaerobic power output, enhanced vasodilation, and increased ATP production in trained individuals [94,95]. These vascular mechanisms are consistent with those described for hesperidin in the cardiovascular section of this review, supporting its inclusion in Table 3 under cardiovascular health. The sports performance context in which these trials were conducted should nonetheless be considered when interpreting the translational relevance of these findings for broader cardiovascular populations.

Weight management is a key application area, with the global weight-loss supplement market valued at USD 33.14 billion in 2024 and projected to grow at a 14.17% CAGR through 2030. Approximately 15% of U.S. adults have used such supplements [96]. Morosil^TM^ (BIONAP), standardized to 2.2% hesperidin/narirutin and 0.9% anthocyanins from non-compliant moro oranges, targets body mass index (BMI), body composition, and fat loss. Its clinical evidence base comprises two trials: an initial pilot study in 30 overweight individuals over 12 weeks, and a larger, more robust 24-week randomized controlled trial involving 180 participants [97,98]. In the latter, participants receiving Morosil^TM^ supplementation exhibited significantly greater improvements compared to placebo, including reductions in body mass, waist and hip circumferences, fat mass, and visceral and subcutaneous fat distribution, without significant changes in liver toxicity markers or cardiometabolic parameters. Together, these studies provide a progressively stronger evidence base supporting the role of OP-derived extracts in body composition management. Sinetrol^TM^ combines a natural blend of orange and grapefruit extracts standardized to >40% total flavanones (mainly hesperidin) with guarana as a source of caffeine. It is important to note that Sinetrol^TM^ is a multi-fruit, multi-ingredient formulation, and the reported clinical effects cannot be attributed exclusively to OP-derived compounds, given the potential contributions of naringin from grapefruit and the thermogenic effects of guarana-derived caffeine. These findings are therefore presented as illustrative of the broader OP-derived polyphenol ingredient. Clinical trials conducted in Caucasian and Asian populations demonstrated that daily supplementation with 900 mg/day for 12 weeks significantly reduced body weight, abdominal fat mass, and waist and hip circumferences, and decreased inflammatory and oxidative stress markers [99,100,101,102]. Moreover, a 16-week RCT demonstrated that Sinetrol^TM^ supplementation improved multiple cardiometabolic markers and gut microbiota in overweight and obese adults, including increased HDL cholesterol, decreased LDL cholesterol, reduced glycated hemoglobin levels, and lower circulating leptin concentrations. Notably, Sinetrol^TM^ also modulated gut microbiota composition, increasing beneficial butyrate-producing bacterial genera such as Eubacterium ruminantium and Ruminococcaceae NK4A214 group, effects that were associated with reductions in visceral adipose tissue and improvements in resting energy expenditure [102].

Despite these promising developments, critical appraisal of the available evidence is warranted. A substantial proportion of clinical studies rely on small sample sizes, short intervention periods, and populations with mild or no cardiometabolic risk, limiting generalizability. Variability in extraction methods, compound standardization, and the use of multi-component formulations complicates attribution of observed effects specifically to upcycled OP ingredients. Furthermore, differences in bioavailability, metabolism, and food matrix interactions contribute to discrepancies between preclinical and clinical findings. Notably, pectin and carotenoids derived from OP remain entirely absent from the commercial clinical evidence base, representing a significant gap given their promising preclinical profiles. Future research should prioritize well-designed, long-term randomized controlled trials using standardized and well-characterized OP ingredients, with clearly defined composition, clinically relevant dosages, and rigorous assessment of bioavailability and long-term safety.

**Table 3:** Summary of commercial orange-derived ingredients: composition and health benefits supported by clinical evidence.

Upcycled OP Ingredient	Product Name	Marketing and Positioning	Main BCs	Source	Posology (mg/Day)	Claimed Health Benefits	Clinical Evidence (References)
**Polyphenol-rich extract**	Microbiomex^TM^	Gut health Prebiotic effect	>80% hesperidin and 5% naringin	Immature oranges and grapefruit (94:6)	400	Contribute to gut barrier strengthening and reduction in gut inflammation. Contributes to microbiota modulation and SCFA production.	↑ Butyrate/SCFA ratio (*p* = 0.022) ↓ Fecal Calprotectin (*p* = 0.058) ↑ *Roseburia* spp. (*p* = 0.049) [62]
	Actiful^TM^	Healthy aging Vitality Energy	>65% hesperidin and pomegranate extract	Immature orange and pomegranate	700	Enhance energy and vitality in mild-aged individuals. Support mental wellness.	↑ handgrip strength (*p* = 0.019) ↑ thinking, memory, learning, and concentration facets (*p* = 0.042) ↓ plasma malondialdehyde (oxidative stress marker) (*p* = 0.033) ↓ methylglyoxal (key factor in cellular aging) by 9.8% [90,91]
	Watts’up^TM^	Power Endurance Strength	>90% hesperidin (of which S-isomer > 60%)	Immature oranges	400	Sports performance booster. Increases ATP production. Increases vasodilation.	↑ aerobic power in athletes (*p* < 0.05) ↓ VO_2_/power ↑ average anaerobic power output in moderately trained individuals (*p* < 0.001) [94,95]
	Cordiart^TM^	Cardiovascular health	90% micronized hesperidin		500	Decrease Blood Flow and Plaque formation Decrease lipid accumulation	↓ endothelial inflammation (E-selectin, VCAM-1 and ICAM-1) ↓ systolic and diastolic blood pressure (*p* < 0.05) ↑ arterial flexibility [66]
	Cardiose^TM^	Cardiovascular health and sports performance	>85% hesperidin (S-isomer)	OP	500	Increases arterial flexibility Reduces plaque formation Decrease inflammation Sports performance booster. Improve antioxidant capacity	↑ anaerobic performance (power, speed, energy) in amateur cyclists (*p* < 0.05) ↑ FTP and max power (*p* = 0.042) ↑ catalase activity, ↓ TBARS [92,93]
	Morosil^TM^	Weight management	2.2% hesperidin/narirutin + 1.0% hydroxycinnamic acids + 0.9% anthocyanins	Juice from non-compliant Moro oranges	400	Support optimal BMI and body composition Support optimal waist and hip circumference	↓ body weight, BMI, waist and hip circumference (*p* < 0.05) in overweight healthy ⊣ adipogenesis [97,98]

Abbreviations: FTP—functional threshold power; SCFA—short-chain fatty acids; BMI—body-mass-index; ↑ Increase; ↓ decrease; ⊣ inhibition.

## 5. Conclusions and Future Directions

This scoping review systematically mapped the evidence on the health-promoting potential of upcycled OP ingredients, mainly flavonoids, pectin, carotenoids, and EO, for GI and cardiometabolic health, drawing on more than 84 in vitro and preclinical studies, as well as 14 completed clinical trials. Among these, polyphenol-rich extracts standardized to hesperidin are the most extensively studied and clinically validated, with consistent evidence across preclinical and human trials supporting effects on gut microbiota modulation, postprandial glycemic response, lipid profiles, blood pressure, and systemic inflammation. Commercial ingredients such as MicrobiomeX^TM^ and Morosil^TM^ exemplify the translational potential of these BCs, from mechanistic evidence to real-world applications, though it is acknowledged that several supporting clinical studies were conducted or funded by ingredient manufacturers, and that some formulations derive from non-standard orange fractions, which warrants cautious interpretation of their outcomes.

In contrast, other upcycled OP ingredients remain substantially underexplored at the clinical level. Pectin, despite robust preclinical evidence supporting its prebiotic activity, modulation of SCFA production, and regulation of glucose and lipid metabolism, has not yet been investigated in dedicated clinical trials as a nutraceutical ingredient derived from OP. Similarly, carotenoids and EO, while demonstrating promising antioxidant, antimicrobial, and anti-inflammatory properties in in vitro models, lack in vivo and clinical validation. This asymmetry in the evidence base, where flavonoids dominate clinical research while other OP fractions remain at the preclinical stage, represents a key limitation of the current literature and a priority area for future investigation.

Current clinical evidence is further constrained by methodological heterogeneity, including small sample sizes ranging from 12 to 111 participants, short intervention periods from acute designs to 12-week protocols, and recruitment predominantly of healthy adults or mildly at-risk individuals, limiting generalizability to clinical populations with established chronic disease. Future research should prioritize large-scale, long-term clinical trials in diverse populations, validate underexplored OP ingredients such as pectin and carotenoids, and employ advanced analytical tools including metagenomics and metabolomics to elucidate microbiota-mediated mechanisms. Comparative studies against conventional fiber or polyphenol sources and investigations into bioavailability and delivery strategies will be critical for positioning OP ingredients as credible, multifunctional solutions within established health strategies. 

This review bridges mechanistic evidence with practical applications, highlighting the role of OP upcycling in advancing sustainable nutrition within the circular bioeconomy. Strengthening the clinical evidence base will not only unlock the full health potential of upcycled OP ingredients but also contribute to broader global strategies for non-communicable disease prevention and environmental sustainability.

## Data Availability

No new data were created in this study. All data analyzed were obtained from publicly available sources, including peer-reviewed publications indexed in Scopus and registered clinical trials available at ClinicalTrials.gov. The information supporting the findings of this review is included within the article.

## Appendix A

**Table A1:** Health-related keyword categories combined with the primary search query used to identify studies on bioactivities of upcycled OP ingredients.

Category	Keywords/Boolean Terms
**Antimicrobial activity**	“antimicrobial” OR “antiviral” OR “antifungal” OR “antibacterial” OR “chronic respiratory diseases”.
**Antioxidant activity**	“antioxidant” OR “oxidation” OR “oxidative stress” OR “peroxidation” OR “free radicals” OR “Reactive oxygen species” OR “ROS” OR “catalase” OR “Superoxide dismutase” OR “Glutathione”
**Anti-inflammatory activity**	“anti-inflammat*” OR “inflammat*” OR “Cytokines” OR “Interleukins” OR “Cyclooxygenase” OR “Prostaglandins” OR “Immunomodulation” OR “Macrophages” OR “Neutrophils”
**Gut modulation**	“inflammatory bowel disease” OR “irritable bowel syndrome” OR “gut homeostasis” OR “gut” OR “gut microbiota” OR “intestinal dysbiosis” OR “microbiota dysbiosis” OR “prebiotic” OR “probiotic” OR “colitis” OR “anti-colitis activity” OR “microbial interaction” OR “gastrointestinal disease” OR “Crohn’s disease” OR “ulcerative colitis” OR “Gut Microbiome”
**Cardiometabolic health**	“Hepatic” OR “Hepatoprotection” OR “hepatoma” OR “liver cancer” OR “Hepatic Lipid” OR “fatty liver” OR “Hepatitis” OR “liver disease*” OR “cirrhosis” OR “Hepatocyte” OR “Alanine Aminotransferase” OR “Aspartate Aminotransferase”
	“Obesity” OR “triglycerides” OR “antilipidemic” OR “cholesterol” OR “anti-obesity” OR “adipolysis” OR “Lipolysis” OR “Dyslipidemia” OR “Hypercholesterolemia” OR “Hypertriglyceridemia” OR “Adipose Tissue” OR “Lipolytic” OR “Adipocyte”
	“diabetes” OR “antidiabetic” OR “anti-diabetic” OR “glucose” OR “glucosidase activity” OR “hypoglyce*” OR “hyperglyce*” OR “glycem*” OR “Insulin”
	“anti-atherogenic” OR “atherosclerosis” OR “cardioprotective” OR “Vasoprotective” OR “arterial pressure” OR “anti-hypertensive” OR “hypertension” OR “angiotensin converting enzyme” OR “Blood Pressure” OR “Hypertensive” OR “ACE” OR “Ischemia” OR “Vasoconstriction” OR “heart” OR “Cardiovascular” OR “endothelial function”
	“metabolic” OR “non-communicable diseases”
**Antineoplastic**	“cancer” OR “anti-cancer” OR “anti-tumor” OR “Chemopreven*” OR “anti-proliferative” OR “chemoprotective” OR “colorectal cancer” OR “colon cancer” OR “gastric cancer” OR “anti-tumoral” OR “Cell Proliferation” OR “neoplastic*”
